# Carbon Monoxide and Prokaryotic Energy Metabolism

**DOI:** 10.3390/ijms26062809

**Published:** 2025-03-20

**Authors:** Vitaliy B. Borisov, Elena Forte

**Affiliations:** 1 Belozersky Institute of Physico-Chemical Biology, Lomonosov Moscow State University, Leninskie Gory, 119991 Moscow, Russia; 2 Faculty of Bioengineering and Bioinformatics, Lomonosov Moscow State University, Leninskie Gory, 119991 Moscow, Russia; 3 Department of Biochemical Sciences, Sapienza University of Rome, I-00185 Rome, Italy; elena.forte@uniroma1.it

**Keywords:** redox enzyme, terminal oxidase, cytochrome, heme, respiratory chain, enzyme inhibition, membrane protein, molecular bioenergetics

## Abstract

Carbon monoxide (CO) plays a multifaceted role in both physiology and pathophysiology. At high levels, it is lethal to humans due to its tight binding to globins and cytochrome *c* oxidase. At low doses, CO can exhibit beneficial effects; it serves as an endogenous signaling molecule and possesses antibacterial properties, which opens up possibilities for its use as an antimicrobial agent. For this purpose, research is in progress to develop metal-based CO-releasing molecules, metal-free organic CO prodrugs, and CO-generating hydrogel microspheres. The energy metabolism of prokaryotes is a key point that may be targeted by CO to kill invading pathogens. The cornerstone of prokaryotic energy metabolism is a series of membrane-bound enzyme complexes, which constitute a respiratory chain. Terminal oxidases, at the end of this chain, contain hemes and are therefore potential targets for CO. However, this research area is at its very early stage. The impact of CO on bacterial energy metabolism may also provide a basis for biotechnological applications in which this gas is present. This review discusses the molecular basis of the effects of CO on microbial growth and aerobic respiration supported by different terminal oxidases in light of recent findings.

## 1. Introduction

**Figure 1:**
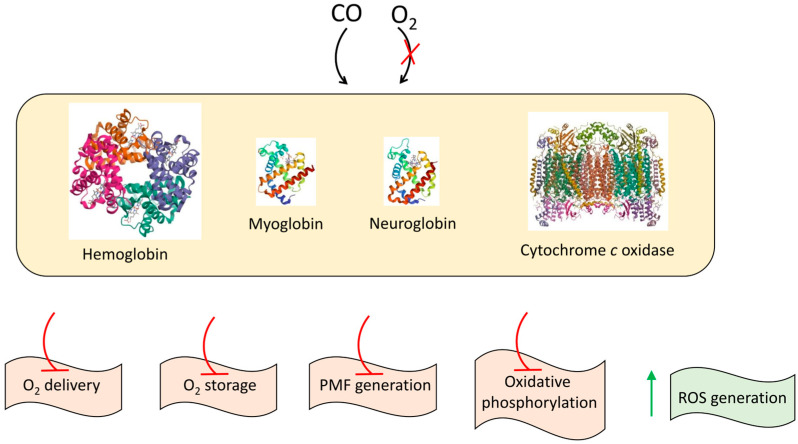
Proposed mechanisms for CO poisoning. The most notable targets of CO include hemoglobin (PDB 1GZX), myoglobin (PDB 3RGK), neuroglobin (PDB 1OJ6), and cytochrome *c* oxidase (PDB 1V54).

It has long been known that carbon monoxide (CO), a nonirritating, colorless, odorless, and tasteless gas, can be lethal to mammals at high levels. The mechanisms of CO poisoning include the tight binding of the gas to hemoglobin, myoglobin, neuroglobin, and the mitochondrial cytochrome *c* oxidase (Figure 1). As a consequence of the reaction between CO and ferrous hemoglobin, carboxyhemoglobin rapidly reaches toxic levels that impede oxygen delivery to tissues [1,2]. The interaction of CO with myoglobin prevents the storage of oxygen. Carboxymyoglobin and carboxyneuroglobin can be a source of extravascular CO storage impacting the severity of CO poisoning [1]. The binding of CO to the fully reduced binuclear active site of cytochrome *c* oxidase interferes with the binding of the substrate, O_2_, to the site [3]. The resulting inhibition of the oxygen reductase activity by CO [4,5] blocks the function of the entire respiratory chain, impairing proton motive force (PMF) formation and ATP synthesis through oxidative phosphorylation. The inhibition of aerobic respiration by CO also triggers the generation of reactive oxygen species (ROS). Excessive levels of ROS become pathological and exacerbate CO poisoning [6].

Surprisingly, when produced in low amounts, CO promotes a wide array of beneficial effects [7]. CO, along with two other biologically active gases, nitric oxide (NO) and hydrogen sulfide (H_2_S), constitute the ‘gaseous triumvirate’ by serving as endogenous signaling molecules [8,9]. As compared to NO and H_2_S, CO has limited chemical reactivity. CO interacts with transition metal ions having specific redox states. The most typical molecular targets are hemoproteins in which CO binds to Fe^2+^ of the heme group. Among such hemoproteins are not only globins and cytochrome *c* oxidase but, for instance, also soluble guanylate cyclase [10], cystathionine β-synthase [11], neuronal PAS domain protein 2 [12], the K_ATP_ channel [13], and cytochrome P450 [14]. There are also nonheme targets of CO, such as Zn^2+^-dependent metalloproteinases [15] and Ca^2+^-dependent potassium channels [16]. CO plays a role in the regulation of circadian clock machinery [17], carotid body activity [18], and insulin secretion [19]. CO appears to exert anti-inflammatory, antiapoptotic, antihypertensive, anticancer, antidiabetic, antimalarial, and antibacterial effects and contributes substantially to protection against ischemia–reperfusion injury, delayed graft function, organ injuries, and sickle cell disease [20]. In plants, CO is also involved in different biological processes, acting as a compound with hormonal effects. It affects seed germination, root development, stomatal closure, and enhances plant abiotic stress resistance [21]. CO also plays a role in the symbiotic relationship between the mammalian host and microbiome. There is growing evidence that CO mediates multidirectional communication between the host and microbes. CO was shown to affect the host immune response [20]. The amplification of the host innate responses by the generated CO enhances the ability of macrophages to clear the pathogen [22,23]. For example, CO increases the survival of mice following the onset of peritoneal sepsis partly through systemic enhancement of autophagy [24,25,26].

**Figure 2:**
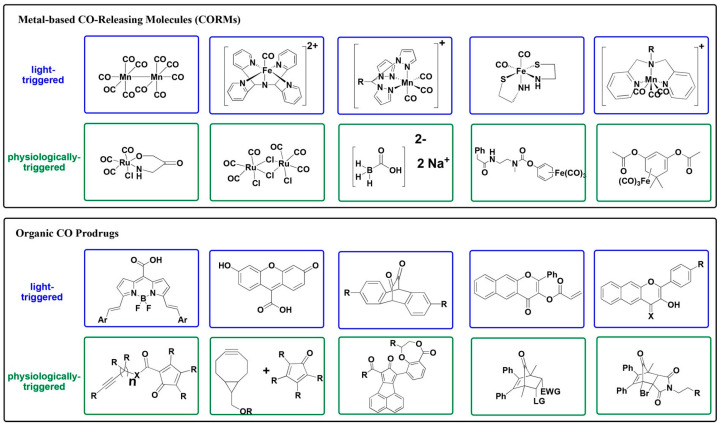
Structures of metal-based CO-releasing molecules and metal-free organic CO prodrugs. Reprinted with permission from Hopper et al. [20]. Copyright © 2020 American Chemical Society.

The fact that CO in small doses demonstrates antimicrobial and anti-inflammatory properties opens up possibilities for its use as a therapeutic agent for disease control. However, controlled CO administration is a challenging issue. The use of CO gas under clinical conditions is not practical. Due to the limited solubility of CO in body fluids, a patient would need to inhale a high concentration of CO, but the delivery of gaseous CO cannot be precisely controlled, and overexposure of body tissue to CO can be toxic [27]. A way to solve the problem is to use biocompatible molecules which can release CO only when triggered by internal or external factors. This includes the development of pharmacologically effective prodrugs referred to as ’carbon monoxide-releasing molecules’ (CORMs), which can supply a human body with CO in well-regulated doses [27,28,29,30,31,32,33,34,35]. CORMs were developed using metals such as ruthenium, iron, molybdenum, and manganese. Metal-based CORMs, as well as metal-free organic CO prodrugs, can be light-triggered and physiologically triggered [20] (Figure 2). The CO prodrugs refer to different structural classes and possess diverse delivery properties, including tunable CO release rates, triggered release (can be activated by endogenous ROS, esterase, and/or changes in pH), mitochondria-targeting, and delivering more than one payload when a single prodrug is used [20]. It should, however, be noted that the widely used Ru-containing CORM-3 appears to reveal cytotoxic effects due to a thiol-reactive Ru(II) ion and releases little CO [36]. Therefore, care should be taken when metal-based CORMs are used. As to organic CO prodrugs, the side products from CO release have to be assessed in control experiments [20]. Finally, the use of hydrogel microspheres which can generate CO gas in situ and thus disrupt bacterial respiration and eliminate bacterial biofilms also sounds promising [37].

**Figure 3:**
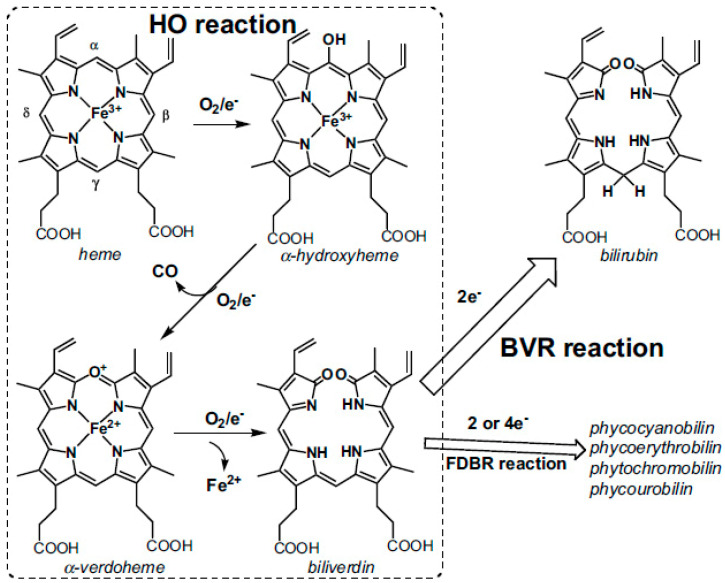
Endogenous CO is a byproduct in the reaction catalyzed by heme oxygenase (HO), in which heme (iron-protoporphyrin IX) is cleaved to produce an open-chain tetrapyrrole, biliverdin IXα, via three steps. Biliverdin IXα is then reduced to bilirubin IXα by biliverdin reductase (BVR). In photosynthetic organisms, biliverdin IXα can be reduced by ferredoxin-dependent bilin reductases (FDBR) to produce pigments, such as phycocyanobilin, phycoerythrobilin, phytochromobilin, and phycourobilin. Reprinted from Sugishima et al. [38] under the terms of the Creative Commons Attribution 4.0 International Public License.

In eukaryotes, CO is generated endogenously mainly from heme oxygenase-mediated degradation of heme [9]. Heme oxygenase forms a 1:1 complex with heme and converts it into biliverdin IXα via three consecutive steps [38] (Figure 3). In the first step, heme is converted into α-hydroxyheme. In the second step, α-hydroxyheme is converted into α-verdoheme that is accompanied by CO release. In the third step, α-verdoheme is converted into biliverdin IXα with concomitant release of ferrous iron. In mammals, the electrons needed for this monooxygenase reaction are supplied by NADPH-cytochrome P450 oxidoreductase. Biliverdin IXα is then reduced to bilirubin IXα by biliverdin reductase using NAD(P)H as a reducing agent [38] (Figure 3). In prokaryotes, CO is produced by homologs of eukaryotic heme oxygenases and analogous enzymes operating via alternative CO-producing mechanisms [20]. Notably, some bacterial pathogens, such as *Bacillus anthracis*, *Escherichia coli* O157:H7, *Listeria monocytogenes*, *Mycobacterium tuberculosis*, *Staphylococcus aureus*, and *Vibrio cholerae*, express heme-degrading enzymes which do not liberate CO upon the catalysis. The lack of CO production probably allows pathogenic microbiota to avoid both self-inflicted toxicity and the generation of signals which would trigger the host immune response [20].

**Figure 4:**
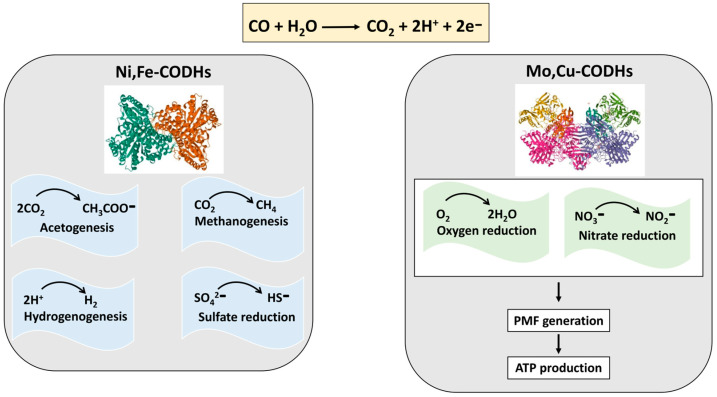
CO oxidation catalyzed by anaerobic CO dehydrogenases, Ni,Fe-CODHs, (PDB 3B52) and aerobic CO dehydrogenases. Mo,Cu-CODHs, (PDB 1N63) fuel different metabolic processes in prokaryotes.

Prokaryotes are capable of using CO as a source of energy and carbon. Microorganisms which can use CO as an energy source to support their growth are called ‘carboxydothrophs’ [39] or “CO oxidizers” [40]. This is possible thanks to its extremely low redox potential, E^0′^ (CO/CO_2_) = −520 mV. CO oxidation can be coupled with the reduction of various electron acceptors and drive different metabolic pathways, including energy conservation and carbon fixation [40]. For this purpose, microbes mostly use CO dehydrogenases (CODHs), which catalyze the reversible oxidation of CO to CO_2_ (Figure 4). CODHs are classified into two distinct phylogenetic and structurally different groups: anaerobic nickel- and iron-containing (Ni,Fe-CODHs) and aerobic molybdenum- and copper-containing (Mo,Cu-CODHs) [41]. The reducing power obtained during CO oxidation by Ni,Fe-CODHs can fuel such processes as acetogenesis, methanogenesis, hydrogenogenesis, and sulfate reduction (Figure 4). Interestingly, Ni,Fe-CODHs couple their function with that of such transmembrane proteins as energy-converting hydrogenase (ECH) in *Carboxydothermus hydrogenoformans* (forming the Ni,Fe-CODH/ECH supercomplex) and energy-converting ferredoxin-NAD^+^ reductase in *Clostridium ljungdahlii*. The coupling allows these machineries to generate PMF or sodium motive force, respectively [40]. In the case of Mo,Cu-CODHs, the reducing equivalents are transferred via cytochrome *b* complex or quinone to a CO-insensitive respiratory chain in which either O_2_ or nitrate serves as a final electron acceptor (Figure 4). The resulting PMF is then used to produce ATP [41].

**Figure 5:**
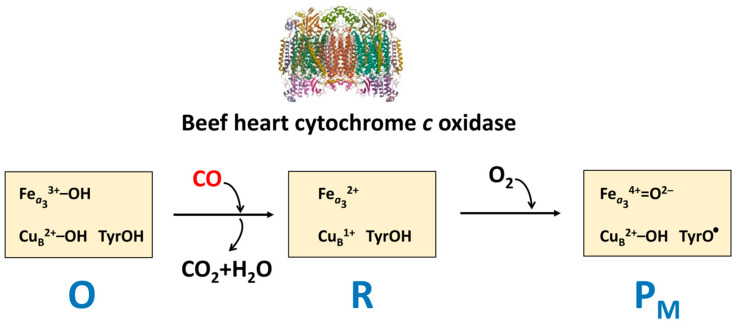
Possible mechanism of CO oxidation catalyzed by beef heart cytochrome *c* oxidase (PDB 1V54). Shown are catalytic intermediates (O, R, P_M_) and the structure of the binuclear *a*_3_/Cu_B_ center for each intermediate.

It is worth mentioning that the mitochondrial (beef heart) cytochrome *c* oxidase can also oxidize CO to CO_2_ [42]. This feature is used to produce the catalytic intermediate P_M_ by bubbling CO into the aerobic solution of the ferric respiratory enzyme [43]. In this reaction, CO presumably donates two electrons to the doubly oxidized (O) *a*_3_/Cu_B_ catalytic site, called the binuclear center (BNC), making it doubly reduced (R). The latter then reacts with O_2_ resulting in the compound P_M_ generation (Figure 5). P_M_ is characterized by the oxidized Cu_B_, the ferryl heme *a*_3_, and a tyrosyl radical assigned to Tyr244 [44,45]. Remarkably, Tyr244 forms a covalent bond with a histidine ligand (His240) of Cu_B_ during enzyme post-translational modification [46]. Because the oxygen reductase activity of cytochrome *c* oxidase is strongly inhibited by CO (an inhibition constant, *K*_i_, of ~0.3 μM [4,5]), such a slow reaction of CO oxidation can hardly decrease significantly the levels of CO within mammals.

CO-metabolizing prokaryotes need to strictly regulate CO metabolic pathways to ensure that gene expression occurs only when CO levels and redox conditions are appropriate [39]. Special heme-based CO-sensing proteins can be used for this purpose. Two CO-dependent transcriptional activators, which regulate oxidative CO metabolism in microbes, were clearly identified as direct CO sensors. These are the CO oxidation activator (CooA) and the regulator of CO metabolism (RcoM) originally found in the phototrophic purple nonsulfur bacterium *Rhodospirillum rubrum* and the soil bacterium *Paraburkholderia xenovoran*, respectively [39]. CooA and RcoM have an N-terminal heme-binding and a C-terminal DNA-binding domain. CO binding to the heme-sensor domain triggers conformational changes which enable protein binding to the target DNA sequence and eventually lead to the upregulation of the transcription of the CODH-coding genes [47,48,49]. Interestingly, in these heme-based CO-sensor proteins, the pathway of allosteric change includes the exchange of an endogenous heme ligand (amino acid residue) and CO, both stable switching configurations being six-coordinate [49].

As CO binds to and inhibits the heme–copper cytochrome *c* oxidase from beef heart mitochondria [4,5], heme-containing prokaryotic oxidases can also be targets for CO. Terminal oxidases of microbial respiratory chains couple the oxidation of reduced cytochrome *c* or quinol by O_2_ to the PMF generation. Studies are underway to acquire information on the CO sensitivity of different types of respiratory enzymes from different prokaryotes. In this review, we discuss the latest data on the influence of CO on the growth and aerobic respiration of *E. coli*, *Mycobacterium smegmatis*, and *Cupriavidus necator* H16.

## 2. Two Superfamilies of Terminal Oxidases

Membrane-bound terminal oxidases are divided into two structurally and evolutionarily unrelated superfamilies: heme–copper oxidases and copper-lacking *bd*-type oxidases, also called cytochromes *bd* [50,51,52,53,54,55,56,57,58,59,60,61,62]. The mammalian genome encodes only one respiratory oxidase, the heme–copper *aa*_3_-type cytochrome *c* oxidase [63,64,65,66,67]. In contrast, the aerobic respiratory chains of prokaryotes may contain enzymes from both superfamilies [68,69,70,71,72,73,74,75,76,77,78,79,80,81,82].

A peculiar feature of heme–copper oxidases is the presence of the BNC where molecular oxygen is reduced to water. The BNC comprises two closely located redox-active groups, a high-spin heme (*a*_3_, *o*_3_, or *b*_3_) and a copper ion named Cu_B_. The catalyzed redox reaction is coupled to the PMF generation through the proton-pumping mechanism [83]. The heme–copper oxidases are classified into three families, A, B, and C, based on structural details, primarily the number and type of proton-transfer pathways [84]. The superfamily members can use cytochrome *c* or quinol as the natural electron donor. Cytochrome *c* oxidases contain a second copper site called Cu_A_, which is mixed-valence and binuclear, directly accepting electrons from reduced cytochrome *c*. Quinol oxidases lack Cu_A_. In addition, heme–copper oxidases carry a low-spin heme (*a* or *b*) that accepts electrons either from Cu_A_ in cytochrome *c* oxidases or from quinol in quinol oxidases and transfers them to the BNC. The *caa*_3_- and *cbb*_3_-type oxidases have one or more covalently bound *c*-type hemes, which serve as additional redox site(s). Heme–copper oxidases can also be associated with other respiratory chain complexes forming supercomplexes [85,86,87,88,89,90].

The *bd*-type oxidases are encoded by prokaryotic genomes only. All biochemically and structurally characterized cytochromes *bd* proved to be quinol oxidases. Interestingly, recent phylogenomic analysis suggests that there are *bd* enzymes which use cytochrome *c* as an electron donor [51]. The active site, where O_2_ is reduced to 2H_2_O, contains a high-spin heme *d* but no copper [91,92,93,94,95,96,97,98,99]. Cytochromes *bd* also have two more hemes, a high-spin *b*_595_ and a low-spin *b*_558_. Heme *b*_558_ mediates electron transfer from quinol to hemes *b*_595_ and *d*. The role of heme *b*_595_ is still not clear; some data suggest that it could perform some of the functions of Cu_B_ [69]. In some cases, heme *d* can be replaced with a *b*-type heme [51,100]. The *bd*-type oxidases produce PMF during the oxygen-reduction reaction but do not pump protons [101,102]. Cytochromes *bd* usually display a very high affinity for O_2_ [103,104]. Phylogenomics identified three families and several subfamilies within the *bd* oxidase superfamily [51]. The earlier classification was based on the size of the hydrophilic region between transmembrane helices 6 and 7 in heme-containing subunit I (often named CydA). This region is a binding domain for quinol oxidation named the Q-loop. Accordingly, cytochromes *bd* can be divided into two families: L (long Q-loop) and S (short Q-loop) [69,105]. It has to be noted that the latter classification is still commonly used.

The main function of most heme–copper oxidases is energy conservation in the form of PMF. The *bd*-type oxidases not only produce PMF but perform other essential physiological functions. In particular, they help prokaryotes to adapt to adverse environmental conditions, such as the presence of some antibiotics, hydrogen peroxide, cyanide, nitric oxide, peroxynitrite, sulfide, and ammonia [106,107,108,109,110,111,112,113]. As cytochromes *bd* are often present in pathogenic bacteria but absent in human mitochondria, they are considered promising protein targets for next-generation antimicrobials [114,115,116,117,118,119,120,121,122,123,124,125,126,127,128].

## 3. Effect of CO on Bacterial Growth and Aerobic Respiration

### 3.1. Effect of CO on E. coli Cell Growth and Aerobic Respiration

**Figure 6:**
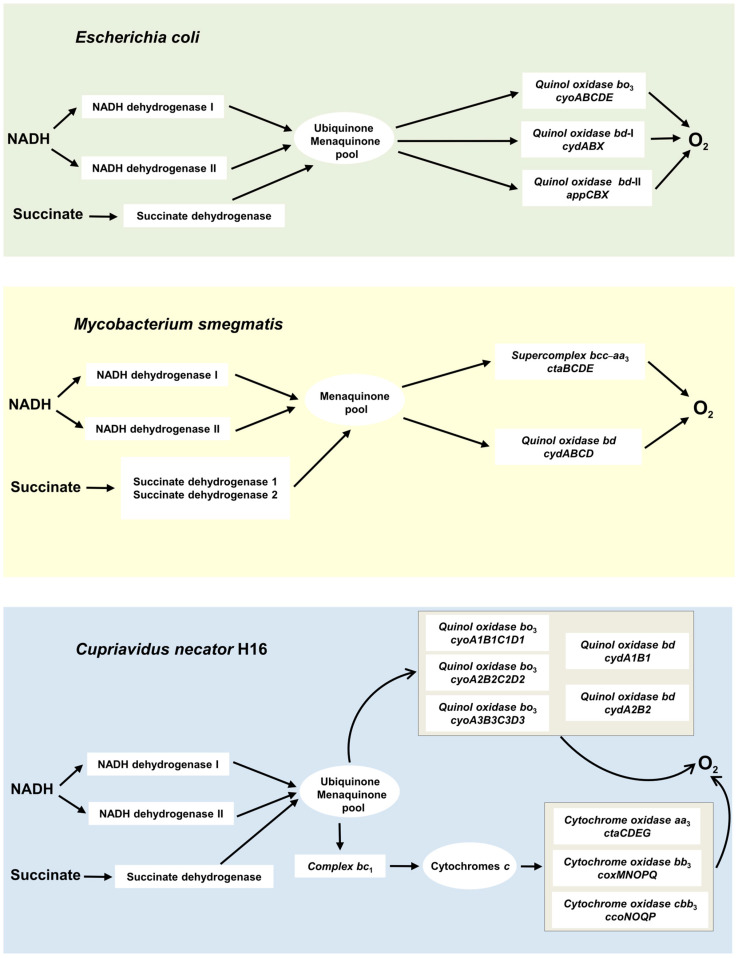
Aerobic respiratory chains of *Escherichia coli*, *Mycobacterium smegmatis*, and *Cupriavidus necator* H16. Arrows indicate direction of electron flow from NADH to O_2_ via the respiratory complexes. Operons, which encode the terminal oxidases, are also shown. Dehydrogenases that use electron donors other than NADH or succinate are not shown for simplicity.

**Figure 7:**
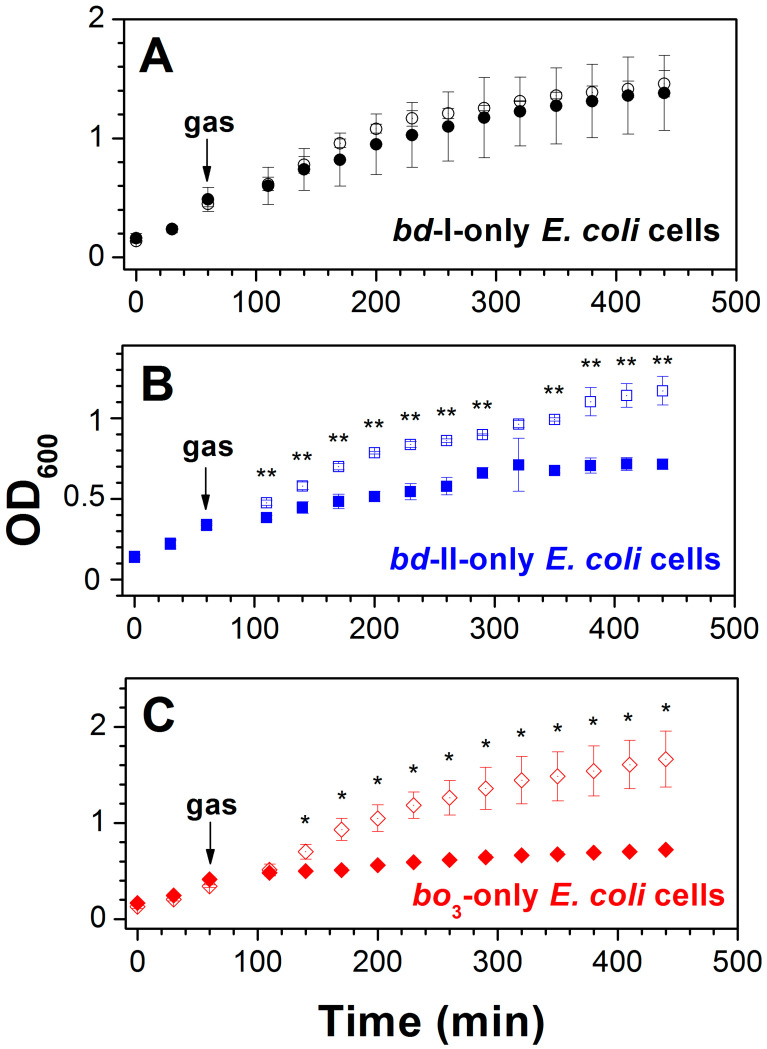
Effect of CO on *E. coli* cell growth. Cell growth of *E. coli* mutant strains expressing either *bd*-I (**A**) or *bd*-II (**B**) or *bo*_3_ (**C**) as the sole quinol oxidase was monitored in the presence of either ~20% CO (‘closed symbols’) or ~20% N_2_ (‘open symbols’). The arrow shows the time (60 min) at which cells were subjected to the gas-flushing treatment for 30 s. Values represent the mean (*n* = 3) ± standard deviation. Asterisks denote statistically significant differences between CO- and N_2_-treated cells (*, *p* < 0,05; **, *p* < 0,01; *t*-test). Reprinted from Nastasi et al. [130] under the terms of the Creative Commons Attribution 4.0 International Public License.

The aerobic respiratory chain of *E. coli* is branched and terminates with three different quinol oxidases, one heme–copper, *bo*_3_, and two *bd*-types, *bd*-I and *bd*-II [101] (Figure 6). The *bo*_3_, *bd*-I, and *bd*-II enzymes are encoded by the *cyoABCDE*, *cydABX*, and *appCBX* operons, respectively. Working with cells and isolated membranes, Nastasi et al. [129,130] found that these oxidases are differently sensitive to CO and consequently can affect *E. coli* growth and aerobic respiration in the presence of the gas under different conditions. The impact of CO on bacterial growth was examined by using three different mutant strains [130]. Each mutant strain expresses only one quinol oxidase, either *bd*-I or *bd*-II, or *bo*_3_. Cell growth of the mutant strains was observed in the presence of either ~20% CO or ~20% N_2_ as a control (Figure 7). The CO addition to the strain possessing *bd*-I as the sole quinol oxidase has little effect on cell growth (Figure 7A). In contrast, the growth of bacterial cells containing either *bd*-II or *bo*_3_ as the only oxidase was severely impaired following the addition of CO, as compared to the control with N_2_ (Figure 7B,C). Therefore, we can conclude that cytochrome *bd*-I promotes *E. coli* growth in the presence of CO, whereas neither cytochrome *bd*-II nor cytochrome *bo*_3_ helps growing bacteria to tolerate CO.

**Figure 8:**
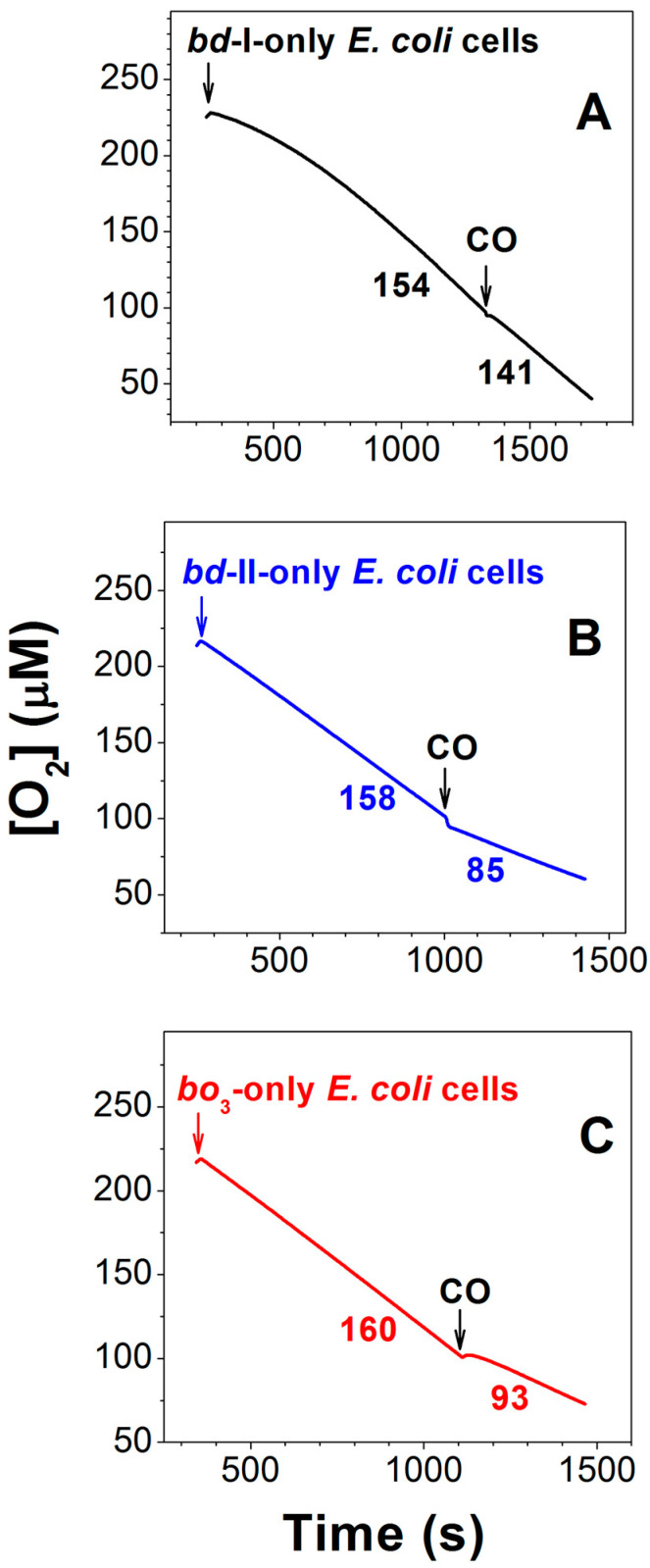
Effect of CO on O_2_ consumption by cell suspensions of *E. coli* mutant strains expressing either *bd*-I (**A**) or *bd*-II (**B**) or *bo*_3_ (**C**) as the only quinol oxidase. Shown are typical experimental traces. A total of 96.3 μM CO was added at [O_2_] = 100 μM. O_2_ consumption rates (nM O_2_/s) measured before and after addition of CO are shown adjacent to each trace. The arrows denote respective additions of bacterial cells or CO. Reprinted from Nastasi et al. [130] under the terms of the Creative Commons Attribution 4.0 International Public License.

The effect of CO on the aerobic respiration of bacterial cells of the three mutant strains was also studied. The respiration was sustained by endogenous respiratory substrates. For this reason, the addition of an exogenous electron donor was not needed. As shown in Figure 8, the inhibition of O_2_ consumption of *bd*-I-only cells by 96.3 μM CO added at 100 μM O_2_ is small (Panel A), whereas it is much stronger in the case of *bd*-II-only and *bo*_3_-only cells (panels B and C). The CO inhibition of cell respiration of the three mutants was studied in detail. CO titration experiments were performed at four different O_2_ concentrations: 50, 100, 150, and 200 μM [129,130]. At all oxygen concentrations tested, the quinol oxidase *bd*-I in cell cultures turned out to be significantly more resistant to CO than the quinol oxidase *bd*-II or the quinol oxidase *bo*_3_. For instance, at 200 μM O_2_, the maximum inhibition percentage at 196.3 μM CO (the maximum [CO] added) in the case of *bd*-I-only cells is 9.7 ± 4.9%, whereas the respective values for *bd*-II-only and *bo*_3_-only cells appeared to be 47.0 ± 6.0% and 39.7 ± 11.5% [130]. It is worth noting that the degree of CO inhibition of each *E. coli* mutant decreases with increasing [O_2_] [130], suggesting competitive inhibition. In other words, in either *E. coli* quinol oxidase, CO competes with O_2_ for binding to the active site under steady-state conditions.

Since the inhibition of O_2_ consumption of *bd*-II-only and *bo*_3_-only mutant cells by CO was significant, it was possible to determine the apparent half-maximal inhibitory concentrations (*IC*_50_) for CO added at different [O_2_] [129,130]. The *IC*_50_ values obtained allowed us to estimate *K*_i_ values for CO, which turned out to be 2.5 ± 0.2 µM and 8.4 ± 0.7 µM for *bd*-II-only and *bo*_3_-only *E. coli* cells, respectively [130].

The effect of CO on the rate of O_2_ consumption of wild-type *E. coli* cells was also studied [130]. In agreement with [131,132], Forte et al. [133] showed that at an early growth phase (low OD_600_) when [O_2_] is high, cytochrome *bo*_3_ is predominantly expressed in wild-type *E. coli*. However, when the bacterial culture reaches high OD_600_ and growth conditions become oxygen-limited, there is a prevalent expression of a *bd*-type oxidase. Consistently, Nastasi et al. [130] reported that aerobic respiration of wild-type cells harvested at high OD_600_ (high *bd*-type cytochrome contents) and low OD_600_ (high *bo*_3_-type cytochrome contents) displays low and high sensitivity to CO, respectively.

In addition, Nastasi et al. [130] examined CO inhibition of O_2_ consumption of membranes isolated from *E. coli* mutant cells. The results appeared to be very similar to those observed with mutant cells. O_2_ consumption of the *bd*-I-containing membranes is relatively resistant to inhibition by CO, while the same reaction catalyzed by both *bd*-II- and *bo*_3_-containing membranes is strongly inhibited by the gas.

Previously, the transcriptomic analysis of wild-type *E. coli* exposed to CO [134] showed that under aerobic conditions the expression of the *cyoABCDE* operon decreases five to ten times, while the expression of the *cydABX* operon increases fourfold. The expression of the *appCBX* operon under the same conditions changes slightly. This is consistent with the data reported by Nastasi et al. [129,130]. Indeed, it sounds logical that after the addition of CO to the wild-type *E. coli* cells, the CO-sensitive cytochrome *bo*_3_ is downregulated, whereas the CO-insensitive cytochrome *bd*-I is upregulated. Cytochrome *bd*-II is not expressed under aerobic conditions, and its upregulation is not required as it is sensitive to the gas.

Thus, the membrane-bound quinol oxidase *bd*-I apparently endows *E. coli* with CO resistance, allowing for aerobic growth and respiration in the presence of the gas at toxic concentrations. It has to be noted that this finding contradicts earlier work [135] showing that O_2_ consumption by the isolated *bd*-I enzyme is sensitive to inhibition by CO. As discussed in [130], the inconsistency likely originated from the difference in the cytochrome *bd*-I environment, detergent micelles in [135] versus a natural lipid bilayer in [129,130].

### 3.2. Effect of CO on M. smegmatis Cell Growth and Aerobic Respiration

The branched aerobic respiratory chain of *M. smegmatis* ends with the cytochrome *bcc*-*aa*_3_ supercomplex, composed of cytochrome *bcc* and the *aa*_3_-type heme–copper cytochrome *c* oxidase, and the *bd*-type quinol oxidase [136,137,138] (Figure 6). It has to be noted that *M. smegmatis* also contains Mo,Cu-CODH to use atmospheric CO as a supplemental energy source. The expression of Mo,Cu-CODH is upregulated when preferred organic energy sources are exhausted. This enhances the long-term survival of the actinobacterium during organic carbon starvation [139]. It is worth noting that CO addition results in enhanced O_2_ consumption in *M. smegmatis* [139]. Electrons derived from CO oxidation by Mo,Cu-CODH may be donated to either of the two terminal oxidases of *M. smegmatis*, thereby coupling Mo,Cu-CODH to the aerobic respiratory chain [140].

Bayly et al. conducted a detailed study of the effect of CO on the growth and aerobic respiration of wild-type and mutant strains of *M. smegmatis* [140]. While studying the growth of the wild-type cells in the presence of 20% CO, they found that *M. smegmatis* is initially inhibited by the gas but grows normally after adapting to CO. Proteomic analysis showed that the CydA and CydB subunits of the *bd*-type quinol oxidase are significantly induced (24- and 4.8-fold) in response to growth in CO, whereas the levels of the *bcc*-*aa*_3_ supercomplex are unaffected. The data on the growth of *M. smegmatis* strains with genetic deletions of the *bcc*-*aa*_3_ supercomplex (Δ*qcrCAB*) and cytochrome *bd* (Δ*cydAB*) in the presence of 20% CO or 20% N_2_ (as a control) were consistent with those with the wild-type strain. It was shown that the growth rate of the Δ*qcrCAB* strain does not differ in the presence or absence of CO, whereas the Δ*cydAB* strain grows significantly slower in CO than in N_2_ [140]. Interestingly, the proteomic analysis showed that in the absence of CO, the Δ*qcrCAB* mutant markedly increases synthesis of CydA (52-fold) and CydB (9.6-fold) as compared to the wild-type, probably to compensate for the loss of the *bcc*-*aa*_3_ supercomplex. To validate the growth phenotypes associated with the Δ*qcrCAB* and Δ*cydAB* mutants, Bayly et al. used a CRISPR interference (CRISPRi) system to independently repress the expression of *qcrC* and *cydA* in a wild-type background strain. It turned out that the growth of *qcrC* and *cydA* knockdown strains in the presence and absence of CO is very similar to that observed for the corresponding knockout strains [140]. Thus, the *bd* enzyme is induced in response to CO and is required for adaptation to growth in CO.

In order to determine the sensitivity of the two different oxidases to CO, Bayly et al. studied amperometrically O_2_ consumption in wild-type, Δqcr*CAB*, and Δ*cydAB* strains by treating respiring cells with a CO-saturated buffer. In the Δ*cydAB* mutant, complete inhibition of O_2_ consumption after CO addition was detected. Inhibition of the wild-type strain appeared to be substantial but less than that for the Δ*cydAB* mutant. Inhibition of the Δ*qcrCAB* mutant by CO was not significant [140]. To validate these findings, the authors also repeated the experiments with the *qcrC* and *cydA* knockdown strains. It turned out that inhibition of O_2_ consumption by CO in these cultures was identical to that observed for their knockout equivalents. Thus, in *M. smegmatis* cell cultures, the O_2_ reductase activity of cytochrome *bd* is resistant to CO, whereas the *bcc*-*aa*_3_ supercomplex is strongly inhibited by the gas [140].

### 3.3. Two bd-Type Terminal Oxidases of C. necator H16 Are Differently Sensitive to CO

The extremely branched aerobic respiratory chain of *C. necator H16* is terminated with eight terminal oxidases, three heme–copper cytochrome oxidases, *aa*_3_, *bb*_3_, and *cbb*_3_, and five quinol oxidases which include three heme–copper *bo*_3_-type and two *bd*-type ones [141,142] (Figure 6). The two cytochromes *bd* are encoded by *cydA1B1* and *cydA2B2* operons [141].

*C. necator H16* can grow heterotrophically using fructose, *N*-acetylglucosamine, gluconate, and fatty acids as electron donors and carbon sources, and O_2_, nitrate, nitrite, or dimethyl sulfoxide as electron acceptors. Under chemolithoautotrophic growth conditions, the bacterium can use CO_2_ as a carbon source, H_2_ as an electron donor, and O_2_ as an electron acceptor. Therefore, *C. necator H16* can be used as a biocatalyst to produce valuable bioproducts, including polymers and potentially chemicals and fuels [143]. For bioproduct production, the bacterium could utilize synthesis gas, an energy-rich feedstock for microbial fermentation. However, the synthesis gas contains not only CO_2_ and H_2_ but also high levels of CO, which the wild-type is unable to metabolize [144].

Wickham-Smith [144] decided to increase the CO resistance of *C. necator H16* through adaptive laboratory evolution. To achieve this, the bacterium was continually subcultured in the presence of CO under different growth conditions. Ten individual cultures that evolved heterotrophically with fructose showed a clear growth advantage over the wild-type strain. A mutation detected in all evolved isolates was a single point mutation upstream of the *cydA2B2* operon. When the mutation was engineered into the parental H16 strain, it enabled faster growth in the presence of CO. The mutation was shown to increase the *cydA2B2* operon expression in *C. necator H16*, possibly by increasing the promoter strength or by enhancing or inhibiting transcription factor binding [144]. The upregulation of the *cydA2B2* transcription appeared to increase CO tolerance under heterotrophic conditions. Notably, expression of *cydA2B2*, but not *cydA1B1*, enables cell growth in the presence of CO. Deletion of *cydA2B2* had a detrimental effect on CO resistance, and plasmid-based expression of *cydA1B1* did not improve CO tolerance [144]. Thus, the data indicate that a *bd*-type quinol oxidase encoded by *cydA2B2* is intrinsically more resistant to CO than other terminal oxidases, including a second cytochrome *bd* encoded by *cydA1B1* [144].

## 4. Possible Mechanisms Underlying Inhibitory Effects of CO on Different Terminal Oxidases

All heme–copper terminal oxidases studied to date appear to be CO-sensitive [4,5,129,130,140]. In contrast, some copper-lacking *bd*-type oxidases are CO-resistant, while others seem to be CO-sensitive [129,130,140,144]. The exact molecular mechanisms underlying the inhibitory effects of the gas on different oxidases remain undefined, but it is clear that CO inhibition is strictly competitive with respect to O_2_. CO directly competes with O_2_ for binding to the enzyme’s catalytic active site. Consequently, in the case of CO-resistant cytochromes *bd*, CO binding to the heme *d* active site should be outcompeted by O_2_. For CO-sensitive oxidases, the situation is reversed: O_2_ binding to the catalytic active site (the *o*_3_/Cu_B_ BNC in cytochrome *bo*_3_, the *a*_3_/Cu_B_ BNC in cytochrome *aa*_3_, or the heme *d* active site in cytochrome *bd*-II) should be outcompeted by CO.

The difference in the affinity of the enzymes for diatomic gaseous molecules may stem from differences in the structural organization of the active sites and their specific environments, including the chemical structure and geometry of the proximal ligand of the heme as well as its distal amino acid residues [145]. Distal residues can stabilize the bound ligand by weak interactions, such as hydrogen bonds, van der Waals interactions, electrostatic effects, and hydrophobic effects, or on the contrary, cause its destabilization due to steric constraints [145,146].

Regarding the big difference in the susceptibility of different oxidases to CO, it should be noted that the rate of CO dissociation from the high-spin heme in the active site of CO-resistant cytochrome *bd*-I is ~260-fold higher than that measured for CO-sensitive bovine cytochrome *c* oxidase (6 s^−1^ [147] vs. 0.023 s^−1^ [148] for the fully reduced enzymes). This is probably one of the main reasons for the difference in CO sensitivity of the enzymes. Indeed, the extremely high CO off-rate would result in a prompt restoration of aerobic respiration supported by the *bd*-I oxidase.

To understand why two oxidases of the same type (*bd*) located in the same bacterium differ in CO sensitivity, it makes sense to look at the structural differences between them. For *bd* oxidases encoded by *cydA1B1* and *cydA2B2* in *C. necator H16*, this cannot be carried out at this stage. They must first be isolated, purified, and then structurally and functionally characterized. However, structures of *E. coli* cytochromes *bd*-I and *bd*-II were reported [92,93,96,97], and some structural differences can be noticed that might affect the sensitivity of the enzymes to CO. (i) The enzymes differ in the number of subunits. Cytochrome *bd*-I contains four subunits, CydA, CydB, CydX, CydY, while cytochrome *bd*-II is composed of three subunits, AppC, AppB, AppX. Interestingly, in the *bd*-I oxidase, CydY shields heme *b*_595_, which is in the high-spin pentacoordinate state, from the lipid bilayer interface. This shielding prevents potential ligands like CO from accessing heme *b*_595_ and, therefore, does not allow them to bind to it [92,93]. Indeed, MCD spectroscopy showed that in the *bd*-I enzyme, heme *b*_595_ is resistant to the binding of some ligands [149,150]. In the CydY-lacking *bd*-II [96,97], direct access of small-molecule ligands to *b*_595_ is not hampered, and CO might bind to the ferrous heme in the steady state. This would slow down electron transfer from heme *b*_558_ to heme *d*, which occurs via heme *b*_595_, resulting in inhibition of the *bd*-II enzyme. (ii) There are data showing that in the *bd*-I and *bd*-II oxidases, different amino acid residues could serve as the heme *d* axial ligand [92,96,97]. If true, this could also contribute to the difference in CO sensitivity between the two cytochromes. (iii) Cytochrome *bd*-I is in the monomeric form, whereas cytochrome *bd*-II incorporated into amphipols is mainly a dimer [96]. (iv) In *E. coli*, each of the two *bd*-type oxidases can associate with other respiratory enzymes, forming supercomplexes. However, for both cytochromes, the composition of such supercomplexes differs. Cytochrome *bd*-I cooperates with formate dehydrogenase and cytochrome *bo*_3_, whereas cytochrome *bd*-II assembles into a supercomplex together with succinate dehydrogenase [86].

**Figure 9:**
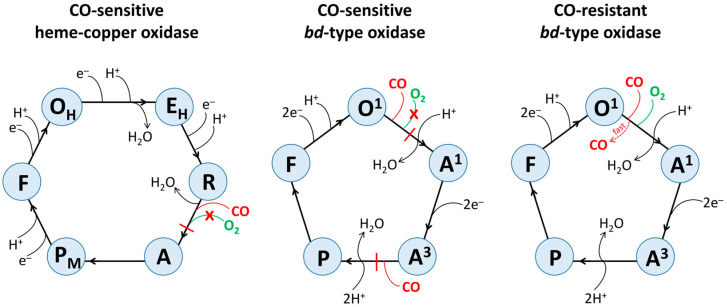
Possible molecular mechanisms for inhibitory effects of CO on the catalytic cycle of different terminal oxidases. Only chemical protons are shown. Pumped protons for heme–copper oxidase are not shown for clarity.

Figure 9 shows possible molecular mechanisms of CO inhibition of the catalytic activity of different terminal oxidases. The catalytic cycle of a heme–copper oxidase includes such intermediates as O_H_, E_H_, R, A, P_M_, and F [55,65,83]. The sequential transfer of two electrons to O_H_ (BNC, consisting of a high-spin heme and Cu_B_, is fully oxidized) leads to sequential formation of E_H_ (BNC is one-electron reduced) and R (BNC is fully reduced). R binds O_2_ to yield A, the primary diatomic oxygen adduct. Then, the O–O bond is cleaved, and A is converted into P_M_ (its structure is mentioned in the Section 1). The transfer of the third electron to BNC re-reduces the tyrosyl radical in P_M_, forming F. The transfer of the fourth electron to BNC regenerates O_H_ and completes the cycle. It is worth mentioning that in the A-family heme–copper oxidases, all transitions except R to A and A to P_M_ are electrogenic and coupled to the transfer of a pumped proton.

It is most likely that in a CO-sensitive heme–copper oxidase, CO binds to Fe^2+^ of a high-spin heme in BNC in the R intermediate producing the Fe^2+^–CO complex (Figure 9). This prevents the binding of O_2_ to the CO-bound heme in BNC, thereby inhibiting the entire catalytic reaction.

The catalytic cycle of a *bd*-type oxidase includes such intermediates as O^1^, A^1^, A^3^, P, and F [102,113] (Figure 9). In O^1^, heme *b*_558_ is reduced, whereas hemes *b*_595_ and *d* are oxidized. In the O^1^-to-A^1^ transition, heme *d* accepts an electron from heme *b*_558_ and binds O_2_. In the A^1^-to-A^3^ transition, two electrons from a quinol molecule reduce hemes *b*_558_ and *b*_595_. In the next A^3^-to-P transition, heme *b*_595_ is oxidized. P is either a true peroxide complex of heme *d* [151] or a ferryl form of heme *d*, in which the O–O bond has been cleaved, with π-cation radical on the porphyrin ring [152] or their mixture [102]. Then in the P-to-F transition, a non-radical form of the ferryl complex of heme *d* is formed, accompanied by the oxidation of heme *b*_558_. The transfer of two electrons from another quinol molecule to F regenerates O^1^ and completes the cycle. The P-to-F and F-to-O^1^ transitions were shown to be electrogenic [101,151,153].

It can be suggested that in a CO-sensitive *bd*-type oxidase, the interaction of the gas with O^1^ stabilizes the electron on heme *d*, resulting in the *d*^2+^–CO complex formation. This prevents the binding of O_2_ to the CO-ligated heme *d*^2+^ and eventually inhibits the enzyme activity (Figure 9). It is also possible that CO binds to heme *b*_595_^2+^ in A^3^, yielding the *b*_595_^2+^–CO complex. This would stabilize heme *b*_595_ in the ferrous state, preventing it from quickly donating an electron to O_2_ bound to heme *d*^2+^ to carry out a concerted four-electron reduction of O_2_ to 2H_2_O.

In a CO-resistant *bd*-type oxidase, CO also reacts with O^1^, producing the *d*^2+^–CO complex. However, in contrast to the CO-sensitive cytochrome *bd*, in this case, thanks to the very high off-rate [147], CO does not bind with high affinity to heme *d*^2+^ and is rapidly ejected from the active site (Figure 9). This results in CO having no significant effect on the catalytic activity of the enzyme. Another factor that possibly contributes to the enzyme’s resistance to CO is the lack of substantial binding of heme *b*_595_^2+^ with the ligand [149,150].

## 5. Concluding Remarks

The mechanisms that prokaryotes use to resist CO are still unclear. CO-insensitive *bd*-type terminal oxidases of prokaryotic respiratory chains likely contribute significantly to these mechanisms. For this reason, when using CO-based antimicrobial medicines, it is important to first determine the CO sensitivity of the terminal oxidases on which the disease-causing pathogenic microorganism relies. If a CO-resistant terminal oxidase is found, CO-based therapy alone will be ineffective and should be combined with selective cytochrome *bd* inhibitors to completely block energy production in the pathogen. In addition, these oxidases may provide a basis for biotechnological applications in which an increased bacterial resistance to CO is needed.

## Data Availability

Data sharing is not applicable.

## Abbreviations

BNC	binuclear center
CO	carbon monoxide
CODH	CO dehydrogenase
CORM	carbon monoxide-releasing molecule
ECH	energy-converting hydrogenase
*IC*_50_	half-maximal inhibitory concentration
*K*_i_	inhibition constant
OD	optical density
PMF	proton motive force
